# Characterizing inflammatory breast cancer among Arab Americans in the California, Detroit and New Jersey Surveillance, Epidemiology and End Results (SEER) registries (1988–2008)

**DOI:** 10.1186/2193-1801-2-3

**Published:** 2013-01-07

**Authors:** Kelly A Hirko, Amr S Soliman, Mousumi Banerjee, Julie Ruterbusch, Joe B Harford, Robert M Chamberlain, John J Graff, Sofia D Merajver, Kendra Schwartz

**Affiliations:** 1 Department of Epidemiology, University of Michigan School of Public Health, Ann Arbor, MI 48109 USA; 2 Center for Global Health, University of Michigan, Ann Arbor, MI 48104 USA; 3 Department of Epidemiology, University of Nebraska Medical Center, Omaha, NE 68198-4395 USA; 4 Department of Biostatistics, University of Michigan School of Public Health, Ann Arbor, MI 48109 USA; 5 Department of Oncology, Wayne State University School of Medicine, Detroit, MI USA; 6 Department of Health and Human Services, Center for Global Health, National Cancer Institute, National Institutes of Health, Bethesda, MD 20892 USA; 7 Dept. of Epidemiology, University of Texas M.D. Anderson Cancer Center, Houston, Texas USA; 8 Department of Radiation Oncology, Cancer Institute of New Jersey - Robert Wood Johnson Medical School, New Brunswick, NJ 08901 USA; 9 Department of Internal Medicine, University of Michigan Medical School, Ann Arbor, MI 48109 USA; 10 Department of Family Medicine and Public Health Sciences, Wayne State University School of Medicine, Detroit, MI USA

**Keywords:** Inflammatory breast cancer, Arab, Race, Hierarchical logistic regression

## Abstract

**Introduction:**

Inflammatory breast cancer (IBC) is characterized by an apparent geographical distribution in incidence, being more common in North Africa than other parts of the world. Despite the rapid growth of immigrants to the United States from Arab nations, little is known about disease patterns among Arab Americans because a racial category is rarely considered for this group. The aim of this study was to advance our understanding of the burden of IBC in Arab ethnic populations by describing the proportion of IBC among different racial groups, including Arab Americans from the Detroit, New Jersey and California Surveillance, Epidemiology and End Results (SEER) registries.

**Methods:**

We utilized a validated Arab surname algorithm to identify women of Arab descent from the SEER registries. Differences in the proportion of IBC out of all breast cancer and IBC characteristics by race and menopausal status were evaluated using chi-square tests for categorical variables, t-tests and ANOVA tests for continuous variables, and log-rank tests for survival data. We modeled the association between race and IBC among all women with breast cancer using hierarchical logistic regression models, adjusting for individual and census tract-level variables.

**Results:**

Statistically significant differences in the proportion of IBC out of all breast cancers by race were evident. In a hierarchical model, adjusting for age, estrogen and progesterone receptor, human epidermal growth receptor 2, registry and census-tract level education, Arab-Americans (OR=1.5, 95% CI=1.2,1.9), Hispanics (OR=1.2, 95% CI=1.1,1.3), Non-Hispanic Blacks (OR=1.3, 95% CI=1.2, 1.4), and American Indians/Alaskans (OR=1.9, 95% CI=1.1, 3.4) had increased odds of IBC, while Asians (OR=0.6, 95% CI=0.6, 0.7) had decreased odds of IBC as compared to Non-Hispanic Whites.

**Conclusions:**

IBC may be more common among certain minority groups, including Arab American women. Understanding the descriptive epidemiology of IBC by race may generate hypotheses about risk factors for this aggressive disease. Future research should focus on etiologic factors that may explain these differences.

## Background

Inflammatory breast cancer (IBC) is an aggressive type of breast cancer with poor prognosis. IBC is characterized by an apparent non uniform geographical distribution in incidence, being more common in North Africa than in other parts of the world. Prior studies have demonstrated that between 1-6% of all breast cancers in the United States are IBC (Taylor & Meltzer 1938; Haagensen 1971; Levine et al. 1985), while the proportion of IBC in Tunisia has been reported as high as 55% (Mourali et al. 1980) with more recent estimates suggesting that IBC represents 5-7% of all breast cancers in Tunisia (Boussen et al. 2008). A population-based study in Egypt established that 11% of all breast cancers there were IBC, which is considerably higher than what is reported in most of the western world (Soliman et al. 2009). In addition to geographical variability in IBC occurrence, studies also suggest that substantial disparities in IBC occurrence may exist by age, race, and socioeconomic status (SES) (Levine et al. 1985; Chang et al. 1998a; Hance et al. 2005). Studies in the U.S. have demonstrated a higher incidence rate of IBC among African American women as compared to White women, and comparable rates among Hispanic and non-Hispanic white women; moreover, a younger mean age of IBC onset among Hispanic women as compared to White and African American women has been noted (Hance et al. 2005; Wingo et al. 2004; Il'yasova et al. 2011). Furthermore, socioeconomics may play an important role in IBC risk, as is evident in the rural predominance of the disease in Tunisia where the SES is generally lower than in urban regions (Mourali et al. 1980; Boussen et al. 2008; Boussen et al. 2010a), and also in the comparison of IBC in North African migrants to France compared with French women living in the same region (Le et al. 2005). A steady decline in IBC cases has been reported appearing in parallel with improved socioeconomic conditions in Tunisia (Boussen et al. 2010b), lending further support for the association between SES and IBC.

Arabic immigrants represent a rapidly growing population in the United States (Zogby 1990; Abraham & Abraham 1983), although the overall size of the Arab American population is highly debated (Jamil et al. 2009). While Arab Americans live in all fifty states, it is estimated that the majority reside in California, Michigan, New York, Florida, and New Jersey (Arab American Institute 2004). Despite the rapid growth of immigrants from Arab nations, little is known about disease patterns among this group because a racial/ethnic category is often not designated for this group, resulting in broad inclusion of Arabs into the White racial category. Previous studies have constructed and utilized surname databases to identify Arab immigrants and to describe relative proportions of different cancer types among this population (Nasseri 2007; Schwartz et al. 2004; Lauderdale 2006). A recent study in the Detroit SEER registry demonstrated increased odds of IBC among Arab Americans as compared to European-Americans (Alford et al. 2009); however, this estimate failed to reach statistical significance, perhaps due to the small sample size.

The aim of this study was to examine the occurrence of IBC among Arab Americans in the California, Detroit and New Jersey SEER registries. These registries have the largest expected Arab American populations and were included to maximize the number of Arab Americans in our sample. Understanding the descriptive epidemiology of IBC in Arab Americans may generate hypotheses about potential risk factors for this aggressive disease.

## Methods

The study population consisted of all women diagnosed with primary invasive breast cancer from 1988–2008 in the SEER population-based cancer registries in Detroit, New Jersey and California. For each case, the following information from routinely collected registry data was obtained: age at diagnosis, race, hormonal receptor status, tumor characteristics, staging, and survival time. The Reporting Recommendations for Tumor Marker Prognostic Studies (REMARK) guidelines were followed in the reporting of the hormonal receptor results (McShane et al. 2005). Assay results for estrogen and progesterone receptor status, prior to neoadjuvant therapy, if available, were abstracted by the SEER registries from the medical record (Johnson & Adamo 2008). Cases where the assay was not performed or was borderline or undetermined were not included in our logistic regression model of marker status by race/ethnicity. The percent of individuals over 25 years of age without a high school diploma within a census tract was also obtained from the SEER registries. We categorized the census-tract level education as high, middle, and low based on tertiles of the overall distribution of this variable in our dataset as follows: High education = <10.85% less than high school graduate; Middle education = >10.85% less than high school graduate and <21.97% less than high school graduate; Low education = >21.97% less than high school graduate. Women 50 years of age and older were considered post-menopausal while those under the age of 50 were considered pre-menopausal. Data were stripped of all personal identifiers, and the analyses were approved by the University of Michigan Institutional Review Board, Wayne State University Human Investigations Committee, the California Protection for Human Subjects Committee, and the Institutional Review Board at the University of Medicine and Dentistry of New Jersey.

Using a validated Arabic name algorithm, we identified Arab American women based on maiden name or surname if maiden name was not available in the National Cancer Institute’s SEER registry data from Detroit, New Jersey and California in 1988–2008. The Arabic name algorithm was created by compiling names from vital statistics records that indicated Arab ethnicity, Arab community group name rosters, and other publicly available name lists. There are over 13,000 surnames on the lists and they have been reviewed multiple times by Arab community members for accuracy. Several quality control measures were used in creating the lists (Schwartz et al. 2004), and a recent telephone validation survey demonstrated that the lists have a 91% positive predictive value [Schwartz et al, in press]. The SEER race codes and Spanish and Hispanic origin variable based on the direct identification component of NAACCR Hispanic Identification Algorithm were utilized to identify the other racial categories in our study. We then compared the proportion of IBC out of all breast cancers and the tumor characteristics and survival time among Arab American, non-Hispanic White (NHW), non-Hispanic Black (NHB), Hispanic, Asian, and American Indian/Alaskan women.

IBC cases were identified using comprehensive coding including ICD-O 8530, which requires pathologic plugging of the dermal lymphatics with tumor emboli, or the extent of disease (EOD) codes EOD-E70 or EOD-E 710–730 or AJCC T4d. This comprehensive case definition of IBC has been utilized in recent publications on IBC from SEER registries (Schlichting et al. 2011; Schairer et al. 2011).

We evaluated differences in the proportion of IBC out of all breast cancer and IBC characteristics by race and menopausal status using chi-square tests or Fisher's exact test for categorical variables and t-tests and ANOVA tests for continuous variables. Log-rank tests were utilized to evaluate differences in IBC survival by race and menopausal status. Logistic regression models were utilized to characterize differences in tumor marker status among the IBC cases by race. We then modeled the association between race and IBC among all women with breast cancer using hierarchical logistic regression models, adjusting for age, tumor marker status, registry and the derived census tract-level education variable. This model accounts for the hierarchical structure and clustering of the data by specifying random effects for the individual-level and census tract-level variables. Confounders were included in the model based upon our prior knowledge. Furthermore, potential confounders that resulted in at least a 10% change-in-estimate criteria between the crude and adjusted measures were included in the model. We tested for interactions between race and each of the characteristics; significant interactions were retained in the model along with their main effects. The hierarchical logistic model was restricted to the years 1999–2008, where tumor marker status information was more regularly reported in SEER. Data analysis was performed using SAS version 9.0 (SAS Institute Inc, Cary, NC); *P*≤.05 was used to determine statistical significance.

## Results

A total of 621,465 female breast cancer cases were included in our study population, of which 9,135 (1.47%) were considered IBC. As shown in Table 1, Hispanic women had the lowest mean age at IBC diagnosis of 52.6 years. Arab Americans (58.5 years) and NHW women (60.1 years) were diagnosed with IBC at older ages compared to the other racial groups in our study. Compared to NHW women, all other racial categories were more likely to be diagnosed with estrogen receptor (ER) negative and progesterone receptor (PR) negative tumors, and were more likely to be diagnosed when pre-menopausal (Table 1), although this trend was the least pronounced among the Arab American women. Arab Americans had the longest mean survival time of 50.5 months, while American Indian/Alaskan natives had the shortest mean survival of 24.8 months (p<.0001). Statistically significant differences in the proportion of IBC out of all breast cancers by racial/ethnic group were evident; 2.91% IBC among American Indian/Alaskan, 2.3% IBC among Hispanics, 2.2% IBC among NHB, 1.7% IBC among Arab Americans, 1.3% IBC among NHW and 1.2% IBC among Asians (Table 2).

**Table 1:** **Inflammatory breast cancer characteristics by race**

		Arab	NHW^a^	NHB^b^	Hispanic	Asian	AI_Al^c^	p-value
		(n=94)	(n=6,035)	(n=1,085)	(n=1,415)	(n=449)	(n=19)	
		n (%)	n(%)	n(%)	n(%)	n(%)	n(%)	
**Estrogen Receptor**^***d***^								
	Positive	38 (42.2%)	2,219 (39.3%)	332 (32.1%)	491 (36.4%)	156 (36.7%)	7 (36.8%)	**0.0070**
	Negative	36 (40.0%)	1,833 (32.4%)	391 (37.7%)	474 (35.1%)	156 (36.7%)	7 (36.8%)	
	Unknown	16 (17.8%)	1,598 (28.3)	313 (30.2%)	384 (28.5%)	113 (26.6%)	5 (26.3%)	
**Progesterone Receptor**^***d***^								
	Positive	23 (25.6%)	1,762 (31.2%)	252 (24.3%)	380 (28.2%)	117 (27.5%)	4 (21.1%)	**0.0003**
	Negative	50 (55.5%)	2,195 (38.8%)	453 (43.7%)	570 (42.2%)	189 (44.5%)	10 (52.6%)	
	Unknown	17 (18.9%)	1,693 (30%)	331 (32%)	399 (29.6%)	119 (28%)	5 (26.3%)	
**Combined Hormonal Status**^**e**^								
	ER+ PR+	20 (27.4%)	1,597 (40.6%)	222 (31.6%)	336 (35.7%)	108 (35.3%)	4 (28.6%)	**0.0002**
	ER+ PR-	17 (23.3%)	538 (13.7%)	98 (14%)	136 (14.4%)	44 (14.4%)	3 (21.4%)	0.0948
	ER- PR+	3 (4.1%)	158 (4.0%)	28 (4%)	42 (4.5%)	9 (2.9%)	0 (0%)	0.8900
	ER- PR -	33 (45.2%)	1,642 (41.7%)	354 (50.4%)	428 (45.4%)	145 (47.4%)	7 (50.0%)	**0.0129**
**Her-2 Receptor**^**f**^								
	Positive	14 (29.2%)	594 (21.9%)	94 (17.9%)	248 (29.9%)	83 (31.1%)	2(18.2%)	**<0.0001**
	Negative	12 (25.0%)	906 (33.4%)	166 (31.7%)	310 (37.3%)	91 (34.1%)	5 (45.5%)	
	Unknown	22 (45.8%)	1,209 (44.6%)	264 (50.4%)	272 (32.8%)	93 (34.8%)	4 (36.3%)	
**Education**^**g**^								
	High	36 (38.3%)	2,233 (37.0%)	183 (16.9%)	198 (14.0%)	128 (28.5%)	3 (15.8%)	**<0.0001**
	Middle	30 (31.9%)	2,021 (33.5%)	249 (23.0%)	262 (18.5%)	127 (28.3%)	8 (42.1%)	
	Low	28 (29.8%)	1,781 (29.5%)	653 (60.2%)	955 (67.5%)	194 (43.2%)	8 (42.1%)	
**Menopausal Status**								
	Pre (<50 yr)	25 (26.6%)	1,565 (25.9%)	389 (35.9%)	648 (45.8%)	162 (36.1%)	8 (42.1%)	**<0.0001**
	Post (≥50 yr)	69 (73.4%)	4,470 (74.1%)	696 (64.1%)	767 (54.2%)	287 (63.9%)	11 (57.9%)	
**%No Education**^***g***^		18.6 (15)	17.7 (13)	29.4 (18)	35.2 (21)	23.7 (17)	28.8 (18)	**<0.0001**
**Age at diagnosis**		58.5 (11.7)	60.1 (14.6)	56.2 (14.5)	52.6 (14)	54.0 (12.5)	54.8 (12.7)	**<0.0001**
**Survival (months)**		50.5 (51.3)	47.6 (49.3)	33.2 (37.5)	43.1 (44.2)	49.7 (47.4)	24.8 (25.7)	**<0.0001**

^a^*NHW*=Non-Hispanic White.

^b^*NHB* = Non-Hispanic Black.

^c^*AI_Al* = American Indian/Alaskan native.

^d^ Sample size for ER and PR for cases diagnosed 1990–2008 only (NHW=5650, NHB=1036, Hispanic=1349, Arab=90, Asian=425, AI_Al=19).

^***e***^ Combined hormonal status based only on non-missing data from 1990–2008 (sample size for NHW = 3935, NHB=702, Hispanic =942 , Arab=73, Asian=306, Al_AI=14).

^***f***^ Sample size for HER2 for cases diagnosed 1999–2008 (NHW=1209, NHB=524, Hispanic=830, Arab=48, Asian=267, Al_AI=11).

^***g***^% No Education (25 years of age or older without a high school diploma) and Education (tertiles of distribution) based on census tract-level information.

*ANOVA test for continuous variables, chi-sq for categorical (or Fisher's exact test for cell counts <5), log-rank test for Survival.

**Table 2:** **Proportion of IBC out of all breast cancers by race and registry**

Race	All Breast Cancer	IBC	%IBC	p-value
All Races combined	621,465	9,135	1.47%	-
NHW	462,717	6,035	1.30%	**<0.0001**
NHB	49,980	1,085	2.17%	
Hispanic	61,062	1,415	2.32%	
Arab-American	5,539	94	1.70%	
Asian	37,085	449	1.21%	
American Indian/Alaskan	652	19	2.91%	
Other	506	6	1.19%	
Unknown	3,924	32	0.82%	
New Jersey Registry	135,764	1,333	0.98%	**<0.0001**
Detroit Registry	60,412	808	1.34%	
California Registry	425,289	6,994	1.64%	

*P-value from chi square test.

In a hierarchical model, adjusting for age, ER, PR, human epidermal receptor 2 (Her2), registry and census tract-level education, Arab-Americans (OR=1.5, 95% CI=1.2, 1.9), NHB (OR=1.3, 95% CI=1.2, 1.4), Hispanics (OR=1.2, 95% CI=1.1,1.3), and American Indians/Alaskans (OR=1.9, 95% CI=1.1, 3.4) all had increased odds of IBC diagnosis as compared to NHW, while Asians had a decreased odds of IBC as compared to NHW (OR=0.6, 95% CI=0.6, 0.7) (Table 3). Interaction terms for race by each characteristic were evaluated in the hierarchical model. The interaction term for race by ER was statistically significant (p<.0001) and was included in the final model. NHW women were less likely to have ER/PR negative tumors as compared to all other racial categories, although this difference was not statistically significant among Arab-Americans and American Indian/Alaskan natives (Table 4). Hispanic (45.8%) and American Indian/Alaskan (42.1%) women had the highest percentage of IBC cases diagnosed in the premenopausal years as compared to the other racial groups, while only 26.6% of Arab American women were diagnosed in premenopausal years (Table 5). Premenopausal IBC cases were more likely to be ER/PR negative, and in the low education category as compared to postmenopausal IBC cases. Further, premenopausal women had a significantly improved mean survival of 96.4 months as compared to 59.2 months among postmenopausal women (p<.0001) (Table 5).

**Table 3:** **Odds ratios (95% CI) for IBC by race**

Race	Crude Model	Age-Adjusted	HLM^a^
Non-Hispanic White	1.0 (ref)	1.0 (ref)	1.0 (ref)
Non-Hispanic Black	1.8 (1.7, 2.0)	1.7 (1.6, 1.8)	1.3 (1.2, 1.4)
Hispanic	1.7 (1.6, 1.8)	1.5 (1.4, 1.6)	1.2 (1.1, 1.3)
Arab-American	1.4 (1.1, 1.8)	1.3 (1.1, 1.6)	1.5 (1.2, 1.9)
Asian	0.9 (0.8, 0.9)	0.8 (0.7, 0.8)	0.6 (0.6, 0.7)
American Indian/Alaskan	2.2 (1.4, 3.5)	2.0 (1.2, 3.2)	1.9 (1.1, 3.4)
Other	0.9 (0.3, 2.3)	0.8 (0.3, 2.0)	1.1 (0.3, 3.8)
Unknown	0.6 (0.4, 0.9)	0.6 (0.4, 0.9)	0.9 (0.6, 1.3)

^a=^Hierarchical logistic regression model adjusted for age, ER, PR, Her2, registry and census tract-level education and included interaction term between race and ER.

**Table 4:** **Odds ratios (95% CI) for marker status at IBC diagnosis by race**

Marker^a^	NHW	NHB	Hispanic	Arab	Asian	AI_Al
ER+PR+	1.0	**0.72 (0.61, 0.84)**	**0.87 (0.76, 0.99)**	0.75 (0.46, 1.2)	0.88 (0.7, 1.1)	0.74 (0.25, 2.2)
ER- PR-	1.0	**1.30 (1.1, 1.5)**	**1.16 (1.02, 1.3)**	1.45 (0.9, 2.2)	**1.28 (1.04, 1.57)**	1.56 (0.6, 4.0)

^a^ ER and PR non-missing data from 1990–2008 only (sample size for NHW = 3935, NHB=702, Hispanic =942 , Arab=73, Asian=306, Al_AI=14).

**Table 5:** **Study population characteristics of IBC cases by menopausal status (age <50 yr vs. >=50 yr)**

		All IBC		Premenopausal IBC		Postmenopausal IBC		p-value
		(n=9,135)		(n=2811)		(n=6,324)		
		n	(%)	n	(%)	n	(%)	
***Race***								
	Non-Hispanic White	6,035	(66.1%)	1,565	(25.9%)	4,470	(74.1%)	**<0.0001**
	Non-Hispanic Black	1,085	(11.9%)	389	(35.9%)	696	(64.1%)	
	Arab-American	94	(1%)	25	(26.6%)	69	(73.4%)	
	Hispanic		1,415	(15.5%)	648	(45.8%)	767	
	Asian	449	(4.9%)	162	(36.1%)	287	(63.9%)	
	American Indian/Alaskan	19	(0.2%)	8	(42.1%)	11	(57.9%)	
	Other	6	(0.07%)	1	(16.7%)	5	(83.3%)	
	Unknown	32	(0.35%)	13	(40.6%)	19	(59.4%)	
***Estrogen Receptor***^***a***^								
	Positive	3,258	(37.9%)	893	(33.8%)	2,365	(39.7%)	**<0.0001**
	Negative	2,909	(33.8%)	1,017	(38.4%)	1,892	(31.7%)	
	Unknown	2,438	(28.3%)	735	(27.8%)	1,703	(28.6%)	
***Progesterone Receptor***^***a***^								
	Positive	2,551	(29.7%)	768	(29.0%)	1,783	(29.9%)	**0.17**
	Negative	3,480	(40.4%)	1,109	(41.9%)	2,371	(39.8%)	
	Unknown	2,574	(29.9%)	768	(29.0%)	1,806	(30.3%)	
***Her-2 Receptor***^***b***^								
	Positive	1,042	(23.6%)	349	(27.3%)	693	(22.1%)	**0.0005**
	Negative	1,496	(33.9%)	426	(33.4%)	1,070	(34.1%)	
	Unknown	1,879	(42.5%)	502	(39.3%)	1,377	(43.8%)	
***Education***^***c***^								
	High	2,801	(30.7%)	814	(29.0%)	1,987	(31.4%)	**<0.0001**
	Middle	2,702	(29.6%)	756	(26.9%)	1,946	(30.8%)	
	Low	3,632	(39.8%)	1,241	(44.2%)	2,391	(37.8%)	
**% No Education**^***c***^		22.1	(17)	24.1	(18.4)	21.2	(16.3)	**<0.0001**
**Age at diagnosis**		58.1	(14.7)	41.7	(5.8)	65.4	(11.2)	**<0.0001**
**Survival time in months**		46.5	(122.2)	96.4	(77.3)	59.2	(61.1)	**<0.0001**

^a^ Sample size for ER and PR for cases diagnosed 1990–2008 only (IBC=8,605, pre-menopause=2,645, post-menopause =5,960)).

^***b***^ Sample size for HER2 for cases diagnosed 1999–2008 (IBC=4,417, pre-menopause=1,277, post-menopause =3,140).

^***c***^ % No Education (25 years of age or older without a high school diploma) and Education (tertiles of distribution) based on census tract-level information.

* ANOVA test for continuous variables, chi-sq for categorical (or Fisher's exact test for cell counts <5), log-rank test for Survival. All statistical tests comparing differences in premenopausal and postmenopausal IBC cases.

## Discussion

This study demonstrated significant differences in the presentation of IBC and the proportion of IBC out of all breast cancers by racial group. Our finding of a younger age at onset of IBC among Hispanic women as compared to NHB and NHW women is consistent with a previous study (Wingo et al. 2004). Almost half of the IBC cases among Hispanic and American Indian/Alaskan natives occurred before the age of 50. While previous studies suggest that IBC rates are similar between non-Hispanic whites and Hispanic women, we found the proportion of IBC out of all breast cancers was significantly higher among Hispanic women as compared to NHW. If the IBC rates are in fact similar between these women, our results may be explained by differences in the trends of non-IBC breast cancer between groups, as non-IBC breast cancer incidence rates have remained stable after declining 7% from 2002 to 2003 (American Cancer Society 2012; DeSantis et al. 2011). These findings highlight the limitation of using proportion of IBC out of all breast cancer instead of IBC incidence rates to evaluate racial disparities. If denominator data were available for the Arab population in the SEER geographic areas, we would have been able to calculate age-standardized incidence rates for the racial groups.The racial disparities in IBC occurrence described in this study may be partially explained by risk factors for IBC that were not adequately controlled for in our analysis. For example, several reproductive factors have been found to be associated with IBC occurrence in previous studies. IBC patients have been reported to have a younger age at menarche and a younger age at first live birth as compared to non-inflammatory breast cancer and non breast cancer patients (Mourali et al. 1980; Chang et al. 1998a; Boussen et al. 2010b; Chang et al. 1998b; Le et al. 2006; Levine 2004). Further, duration of breast feeding exceeding 24 months was found to be significantly associated with IBC in one study (Le et al. 2005). If these reproductive factors are in fact risk factors for IBC and differ by race, as we may suspect (Anderson et al. 2005; Hall et al. 2005), it could possibly explain some of the racial disparities in IBC occurrence observed in our study. In addition to reproductive risk factors for IBC, obesity has been shown to be a risk factor for premenopausal IBC but not for postmenopausal non-IBC in one study (Levine & Venerose 2005), while another study demonstrated that IBC patients had significantly higher BMI than both non-IBC patients and non-breast cancer patients irrespective of menopausal status (Chang et al. 1998b). Finally, we utilized census-tract level information on education as a proxy for socioeconomic status, to account for the contextual effect of living in a community with lower educational attainment, since individual-level education and SES information was unavailable in our dataset. According to 2010 Census information, African-Americans and Hispanics have similar rates of poverty, which are approximately threefold greater than Whites (The US Census Bureau 2010). Without detailed information on reproductive factors, obesity and individual-level SES available in the SEER dataset, we cannot control for these factors in our analysis. Therefore, it is possible that some of the difference in proportion of IBC by race may be explained by residual differences in risk factors that are not accounted for in our study. It has been suggested that the effect of certain risk factors for IBC may differ according to menopausal status (Levine & Venerose 2005). This was apparent in urban–rural differences in IBC cases in Tunisia seen only in premenopausal patients (Mourali et al. 1980), and in obesity as a risk factor for premenopausal women only (Levine & Venerose 2005). Therefore, we stratified our hierarchical model by derived menopausal status to evaluate whether menopause modified the association between race and IBC. Our derived menopausal status variable has been shown to be a robust indicator of actual menopausal status (Phipps et al. 2010; Morabia & Flandre 1992), and has been utilized in several population-based studies on breast cancer (Anderson et al. 2003; Anderson et al. 2004). Stratifying our results for the effect of race on IBC, we found that menopausal status did not significantly modify the effect of race on IBC (data not shown); however, we did find significant differences in disease characteristics between pre-menopausal and post-menopausal IBC cases. The differences in education and hormonal receptor status may provide evidence for differing etiologies for premenopausal and postmenopausal IBC cases, and this should be considered in future research on IBC risk factors. However, it is important to note that we used age as a proxy for menopausal status. Thus, differences in IBC occurrence by menopausal status in our analysis may simply reflect the effect of age and not necessarily an effect of menopause.

Early treatment is critical to improve outcomes for IBC. Our study found improved survival among Arab Americans IBC cases compared to all other racial categories. This finding was also recently reported in non-IBC cases among Arab Americans (Alford et al. 2009). American Indian/Alaskan natives were found to have the shortest mean survival time, and efforts to reach these populations for early treatment of disease should become a priority. We also found improved survival times among premenopausal IBC cases as compared to postmenopausal women, which is not entirely surprising due to the implications of age on survival. These survival disparities need to be addressed and may reflect a lack of early detection, lack of timely and aggressive treatment, and access to care. Without complete treatment information including chemotherapy in our dataset, we are unable to explore these survival differences in more depth in this study.Limitations of this study include a potential for misclassification of Arab women, especially where the maiden name was unavailable. We were unable to assess the magnitude of this potential misclassification bias, as we did not have access to the actual surnames within our dataset. However, we believe that the possibility of misclassification is limited as many Arab ancestry women keep their maiden names upon marriage (Al-Hegelan 1980; Kayyali 2006; Kleffner Nydell 2005), and maiden names are available for a large proportion of the women. Another possible limitation of this study was the lack of information on country of origin or date of immigration to the United States. The Arab American immigrant group is composed of individuals from many diverse Arab nations. Without information on country of origin, we may be missing critical information that could explain disparities in IBC occurrence. We did evaluate the place of birth variable in our dataset, however this variable was missing for 42% of breast cancer cases. Therefore, we were unable to accurately assess this factor in our analysis. Further, we would surmise that immigrants arriving earlier in life would be more likely to experience cancer rates comparable to non-Arab Whites versus immigrants who arrived later (Zogby 1990). Without information on time of immigration, we may be mixing the effect of IBC occurrence between recent Arab immigrants, who maintain certain cultural norms from their countries of origin, with Arab women who have become acculturated to the Western lifestyle after having been born in or living in the U.S. for a considerable amount of time. It would be beneficial to evaluate IBC cancer occurrence by time of immigration among migrant groups in the U.S. in order to understand potential environmental risk factors for the disease. A further limitation could be the use of different laboratories to determine hormonal receptor status in our dataset. Additionally, the hormonal receptor data were not routinely collected during our study period, so we do have to be concerned about missing data for these variables. To overcome this limitation, we restricted our analysis on ER/PR from 1990 forward and on Her2 from 1999 forward, when this information was more regularly reported in the SEER registries. Lack of data on other potentially important covariates including reproductive factors, obesity, individual-level SES, acculturation, and urban/rural status could lead to residual confounding in our analysis. It is also important to consider that our data came from the Detroit SEER, which only includes 3 counties in Michigan, while the California and New Jersey registries are state-wide. This could potentially affect the generalizability of our results if we think that these registries are not representative of the overall population of women with breast cancer in the United States. Finally, this is a purely descriptive analysis and we are unable to draw causal inferences from the results.

Strengths of this study include the use of large-scale population-based SEER registry data, which is considered to be reliable and accurate as it meets International Agency for Research on Cancer (IARC) standards, ensuring a certain degree of data quality and comparability based on a number of factors (Parkin et al. 2003). Further, the IBC case ascertainment definition used in this study is considered valid and is not as conservative as previous studies requiring the pathological diagnosis of IBC. The name algorithm to identify Arab ancestry has been constructed and utilized to describe relative proportion of cancer among this population in previous studies (Nasseri 2007; Schwartz et al. 2004; Lauderdale 2006). Finally, this study is innovative as it maximized the number of Arab Americans represented in the study sample by applying data from California, Detroit, and New Jersey registries.

## Conclusions

Our results suggest that IBC occurrence may be more common among certain minority groups, including Arab American women. With the significant lack of epidemiologic data on IBC, this study represents important progress to our understanding of this rare and aggressive disease. By evaluating racial disparities in IBC occurrence, we hope to generate further hypotheses about potentially modifiable risk factors for IBC. Future research should focus on etiologic factors that may underlie these differences and also examine country of origin and date of immigration to the U.S. to further understand potentially modifiable risk factors for IBC.
